# Analysis of Prostate Cancer Susceptibility Variants in South African Men: Replicating Associations on Chromosomes 8q24 and 10q11

**DOI:** 10.1155/2015/465184

**Published:** 2015-08-12

**Authors:** Pedro Fernandez, Muneeb Salie, Danielle du Toit, Andre van der Merwe

**Affiliations:** ^1^Division of Urology and Tygerberg Hospital, Stellenbosch University, P.O. Box 241, Cape Town 8000, South Africa; ^2^SA MRC Centre for TB Research and DST/NRF Centre of Excellence for Biomedical Tuberculosis Research, Division of Molecular Biology and Human Genetics, Stellenbosch University, P.O. Box 241, Cape Town 8000, South Africa; ^3^Unistel Medical Laboratories, P.O. Box 241, Cape Town 8000, South Africa

## Abstract

Genome-wide association studies (GWAS) have implicated single nucleotide polymorphisms (SNPs) on chromosomes 2p15, 6q25, 7p15.2, 7q21, 8q24, 10q11, 10q26, 11q13, 17q12, 17q24, 19q13, and Xp11, with prostate cancer (PCa) susceptibility and/or tumour aggressiveness, in populations of African, European, and Asian ancestry. The objective of this study was to confirm these associations in South African Mixed Ancestry and White men. We evaluated 17 prioritised GWAS SNPs in South African cases (331 Mixed Ancestry and 155 White) and controls (178 Mixed Ancestry and 145 White). The replicated SNP associations for the different South African ethnic groups were rs7008482 (8q24) (*p* = 2.45 × 10^−5^), rs6983267 (8q24) (*p* = 4.48 × 10^−7^), and rs10993994 (10q11) (*p* = 1.40 × 10^−3^) in Mixed Ancestry men and rs10993994 (*p* = 1.56 × 10^−9^) in White men. No significant associations were observed for the analyses stratified by disease aggressiveness in the individual and the combined population group analysis. The present study demonstrates that a number of known PCa susceptibility variants may contribute to disease susceptibility in South African men. Larger genetic investigations extended to other South African population groups are warranted to confirm the role of these and other SNPs in disease susceptibility.

## 1. Introduction

Prostate cancer (PCa) is the most frequently diagnosed cancer in men in developed countries, with higher incidence rates in the United States of America (USA) in individuals of African ancestry [1]. African American (AA) men have a PCa incidence rate that is 1.6 times that in European American (EA) men and the mortality rate is 2.5 times greater than EA men [2]. In developing countries, recent estimates suggest that PCa is also the leading cancer in terms of incidence and mortality in men from Sub-Saharan Africa (SSA) and the Caribbean [3]. Moreover, underdiagnoses and underreporting have been shown to be widespread in men from SSA and the Caribbean [4], further indicating that incidence and mortality rates may be substantially underestimated in developing countries.

Numerous genome-wide association studies (GWAS) have identified close to 100 PCa susceptibility variants, primarily in populations of European ancestry [5–12], to a lesser extent in AA men [8, 12, 13], and, recently, in African men [14, 15]. Even though many of these associations exceeded or were close to genome-wide significance levels, it remains necessary to confirm these findings in multiple independent populations to rule out false positive results, thereby improving the likelihood that they represent true associations. To this end, several studies have replicated GWAS associations in AA, EA, and Asian men [16–29]. Although GWAS have generally been undertaken to a lesser extent in African descent populations, a recent meta-analysis of 82 PCa susceptibility variants identified primarily in GWAS in European and Asian descent populations generally demonstrated consistent replication of the direction of effects in men with African ancestry [30].

In the present study, we investigated PCa susceptibility single nucleotide polymorphisms (SNPs) in men from two separate South African population groups, one group of mixed Khoisan (San), African, European, and Asian descent and the other of European descent. We report replicating associations with PCa susceptibility for three of the 17 investigated SNPs among Mixed Ancestry (also termed South African Coloured) and White men.

## 2. Materials and Methods

### 2.1. Study Population

The study population comprised 331 Mixed Ancestry and 155 White cases (ethnicity was self-reported for all participants, as previously described) [31]. All cases had no prior cancer at any other site and were recruited from the Division of Urology, Tygerberg Hospital (Cape Town, South Africa). Clinical characteristics including prostate specific antigen (PSA), Gleason score, tumour node metastasis (TNM) stage, age at diagnosis, and other cancer diagnoses were obtained from medical records. All cases underwent radical prostatectomy, transurethral resection of the prostate, or prostatic biopsy and had histologically confirmed PCa. Cases were additionally stratified by risk of developing aggressive PCa using the D'Amico Cancer Risk Group Classification system. Probability of developing aggressive PCa in cases was categorized as “low” (tumour category < T1-2a or PSA ≤ 10 ng/mL or Gleason score < 7) or “high” (tumour category ≥ T2b or PSA > 10 or Gleason score ≥ 7) (“high” was defined here as the combination of intermediate + high risk).

Controls were selected among subjects admitted to Tygerberg Hospital for routine PSA examinations or benign prostatic hyperplasia (BPH) and comprised of 178 Mixed Ancestry men and 145 White men, matched for age and ethnicity (self-reported), and were from the same geographical region. Inclusion criteria for controls were PSA levels of ≤4.0 ng/mL and a normal digital rectal examination (DRE) or negative biopsy on histology.

All participants were informed and gave written consent to participate in the study and allow their biological samples to be genetically analysed, conducted in accordance with the Declaration of Helsinki. The study was approved by the Stellenbosch University Faculty of Medicine and Health Sciences Human Research Ethics Committee.

### 2.2. SNP Selection and Genotyping

Based on previously reported PCa GWAS and replication studies, we selected 17 SNPs on chromosomes 2p15, 6q25, 7p15.2, 7q21, 8q24, 10q11, 10q26, 11q13, 17q12, 17q24, 19q13, and Xp11. Given the diverse ancestral lineages of the South African population, the SNPs were prioritised for analyses primarily if they had been implicated in PCa susceptibility in GWAS and/or replication studies in European, African, and Asian descent populations. At the time of initial SNP selection, the novel PCa susceptibility variants identified in African men [14, 15] had not yet been reported and were thus not investigated in this study.

Genotyping was performed at the Stellenbosch University Central Analytical Facility (CAF) (Cape Town, South Africa) on the 17 selected SNPs using commercially available Taqman SNP Genotyping Assays (Life Technologies, USA). Quality control (QC) for exclusion from the study for individual samples was a genotype call rate <90%. For all SNPs, genotyping QC was assessed by including DNA duplicates (to an equivalent of 5% duplicates per 384-well plate), and the concordance genotype call rate between samples and duplicates was set at ≥95%. We excluded all SNPs whose genotype frequency departed from Hardy-Weinberg equilibrium (HWE) at *p* < 0.01 in the controls and SNPs that were monomorphic (had a frequency <1%) in our study populations.

### 2.3. Statistical Analyses

Descriptive analyses for discrete traits were carried out using contingency table methods and Fisher's exact tests (FET) or chi-square statistics. Medians were used to summarize continuously distributed traits. We tested the SNPs in the controls for departures of HWE and determined allele and genotype frequencies in cases and controls. For each SNP in each population group, logistic regression models were used to estimate PCa genotype and allelic odds ratios (OR), 95% confidence intervals (CI), and corresponding *p* values adjusted for age (i.e., age at diagnosis for cases and age at ascertainment for controls). We adjusted for age and ethnicity in the combined population logistic regression analysis. Logistic regression was similarly used to estimate OR, 95% CI, and *p* values for cases stratified by disease aggressiveness (“low” versus “high”). All analyses were performed in R, freely available from http://www.R-project.org/. In the present study, we adopted a conservative approach by only considering a Bonferroni corrected *p* value threshold of *p* < 1.47 × 10^−3^ statistically significant (*α* = 0.05/17 SNPs × two population groups). This correction may be considered to be overly conservative [32]; however, because the number of multiple comparisons we performed was relatively small, we opted to use a family-wise error rate approach instead of a less stringent approach [33].

## 3. Results

The median age at diagnoses of PCa cases and the median age of controls were significantly different among the groups; PSA levels were significantly different between the groups, with Mixed Ancestry controls and cases showing higher median levels (Table 1). No significant differences were noted for Gleason score and tumour stage between the two groups, although Mixed Ancestry men showed significantly higher frequency of distant metastasis at the time of diagnosis (Table 1).

For the genotype QC analysis, two cases and one control Mixed Ancestry sample achieved a genotype call rate <90% and were thus excluded, leaving the analysed total of cases at 329 Mixed Ancestry and 155 White and a total number of controls at 177 Mixed Ancestry and 145 White. Additionally, four of the 17 selected SNPs were excluded from further study: rs721048 (2p15) and rs5945619 (Xp11) failed to meet the QC concordance genotype call rate of ≥95%, and Bd11934905 (8q24) and rs7210100 (17q21) were monomorphic in both population groups.

Of the 13 remaining prioritised SNPs, three were significantly associated with PCa after correction for multiple testing in Mixed Ancestry men: rs7008482 (8q24) (OR = 0.41, 95% CI: 0.26–0.62; *p* = 2.45 × 10^−5^), rs6983267 (8q24) (OR = 0.35, 95% CI: 0.23–0.53; *p* = 4.48 × 10^−7^), and rs10993994 (10q11) (OR = 1.56, 95% CI: 1.56–6.08; *p* = 1.40 × 10^−3^) (Table 2). In White men, only rs10993994 (OR = 3.49, 95% CI: 2.28–5.54; *p* = 1.56 × 10^−9^) was significantly associated with PCa after correction for multiple testing (Table 2). In the combined population analysis, the associations for rs7008482, rs6983267, and rs10993994 and PCa susceptibility were statistically significant (Table 2). No significant associations were observed for the case-only analyses stratified by disease aggressiveness (“low” versus “high” risk) in the individual and the combined population group analysis (data not shown).

## 4. Discussion

The present study replicates previously reported associations for rs7008482, rs6983267, and rs10993994, in two different South African population groups. Men from the two population groups reported here have PCa age-standardized incidence rates that are between 1.4 and 2.4 times higher than men from other South African population groups (Mixed Ancestry: 39.1 per 100,000 and White: 52.0 per 100,000 compared to Asian: 28.6 per 100,000 and Black: 21.6 per 100,000) (National Cancer Registry, 2009) [44]. When we compared the age at diagnosis between the two groups, Mixed Ancestry men were shown generally to be diagnosed with PCa at an earlier age than White men (67 versus 70 years). Additionally, Mixed Ancestry men, who have substantial proportions of African ancestry (combination of San, European, Black, and Asian ancestry [45]), showed significantly higher frequency of distant metastasis at the time of diagnosis. A few studies have suggested that men with African ancestry more often present with aggressive disease compared to men of European ancestry [29, 46]. Though highly speculative, our observations may support this view, although our genetic analysis stratified by disease aggressiveness failed to provide supporting evidence.

Most GWAS and replication studies have consistently identified genetic associations with SNPs on chromosome 8q24 [5–8, 10, 11, 16, 19, 20, 22, 23, 25, 26]; two 8q24 SNPs were associated in our study, whereas in another study South African Black men showed associations with the rs6983561 [29]. It has been suggested that multiple, independent polymorphic variants on chromosome 8q24 may produce a common biological mechanism that contributes to disease, or, alternatively, the 8q24 regions may cumulatively influence the regulation of adjacent genes (cis-regulation) or genes on other chromosomes (trans-regulation) [47]. Additionally, it has been suggested that the 8q24 regions harbor previously unannotated microRNAs (miRNAs) and that the miRNA elements in these regions are involved in cis-regulation of distal genes, thereby affecting RNA expression [48]. However, it remains inconclusive why so many studies have shown PCa susceptibility associations to 8q24 given that it is a gene-poor region.

We showed that the SNP rs10993994 was associated with PCa susceptibility across both population groups, as well as in the combined populations. Pomerantz et al. [39] demonstrated that the T-allele of rs10993994 decreased expression in normal tissue of both isoforms of the beta-microseminoprotein gene (*MSMB*), as well as increasing the expression of the five isoforms of the nuclear receptor coactivator 4 gene (*NCOA4*). Furthermore, it was speculated that because rs10993994 is not a susceptibility allele for other cancers, such as colon or breast cancer, the associations observed with this SNP may thus be specific to prostate cancer [39].

In the present study, we opted to use a very conservative approach to reduce the chance of identifying false positive associations. However, we noted SNPs that would conventionally be considered associated with the disease if a less stringent statistical approach was employed. In the White group, rs1048657 (7p15), rs4430796 (17q12), and rs1859962 (17q24) were significant at *p* < 0.05 but not after correction for multiple testing (Table 2). For the Mixed Ancestry, because of the effect of population stratification, we only consider a *p* < 4.1 × 10^−3^ statistically significant (also applied when combining the Mixed Ancestry with other groups) [31]. For the Mixed Ancestry and the combined group, only rs7008482, rs6983267, and rs10993994 were significantly associated after correction for population stratification as well as for multiple testing (Table 2). The individual samples sizes in our study were small; therefore, larger genetic investigations in the population groups investigated here are required to conclusively establish whether or not the selected SNPs play a role in PCa susceptibility.

Our study findings should be interpreted in light of some limitations. We acknowledge that our investigations were only performed on men from the two South African population groups with the highest incidence of PCa and recognize that significant underreporting and underdiagnosis of PCa have been documented in other groups, particularly in South African Black men [49]; thus, future studies would need to be extended to other population groups. Our study sample was relatively small and this may have disadvantaged our power to detect statistical significance. We excluded two SNPs (Bd11934905 and rs7210100) with minor allele frequencies (MAFs) < 1%, because there may not be sufficient power to detect the effect of rare variants due to our sample size; however, larger studies should be undertaken as we cannot disregard them as potential disease-causative SNPs. Population stratification is an additional confounder in case-control association analyses. Daya and colleagues [45] have shown using ancestry informative markers (AIMs) specific for the South African Mixed Ancestry population that genetic associations may change after adjustment for ancestry (previous associations may be abolished or associations may only become apparent after adjustment for ancestry). However, the authors also noted that association results are more likely to be unaffected by adjustment for ancestry if the allele frequencies in the stratified population do not differ markedly from the source populations [45]. When we compared the allele frequencies of the selected SNPs in the Mixed Ancestry to likely source populations in the 1000 Genomes database (African, European, South, and East Asian), the allele frequencies we observed often differed by <5%.

## 5. Conclusions

We demonstrate that a number of reported susceptibility SNP variants may contribute to PCa susceptibility in South African Mixed Ancestry and White men. However, this was a relatively small study and only men from two population groups were investigated. Consequently, larger genetic investigations extended to different South African population groups are warranted to confirm the role of these and other SNPs in PCa susceptibility.

## Figures and Tables

**Table 1:** Clinical characteristics among South African cases and controls.

Variables of interest	Mixed Ancestry		White		*p* value
	Controls (*n* = 178)	Cases (*n* = 331)	Controls (*n* = 145)	Cases (*n* = 155)	
Median age (years)^a^	60	67	64	70	**<0.001**
Median PSA (ng/mL)^b^	0.89	18.4	0.86	11.7	**<0.001**
Gleason ≥ 7^‡^		84/177 (41.7%)		42/96 (43.8%)	0.557
Stage ≥ 2b^‡^		118/283 (41.7%)		39/123 (31.7%)	0.058
Metastasis^‡^		30/252 (11.9%)		4/116 (3.4%)	**0.009**

^a^For each of the respective population groups, *p* < 0.05 when comparing the median age between cases and controls.

^b^For each of the respective population groups, *p* < 0.05 when comparing the median PSA between cases and controls.

^‡^Gleason score, tumour stage, and/or metastases information were missing from the medical records of some of the clinically confirmed prostate cancer cases. Therefore, the numbers and percentages shown are for the available information and not that of the total number of cases included in the study for each population group.

**Table 2:** Summary results for SNP genotyping analysis in South African men.

rs number	Cytoband	In/nearest gene^‡^	Effect allele; frequency (control | case); per effect allele OR (95% CI)^a^; *p* value (adjusted for age)		
			Mixed Ancestry	White	Combined^b^
rs9364554	6q25	*SLC22A3 *	T; (0.13 | 0.11); 0.236	T; (0.20 | 0.21); 0.493	T; (0.17 | 0.15); 0.737

rs10486567	7p15	*JAZF1 *	G; (0.17 | 0.22); 0.098	G; (0.15 | 0.19); 1.89 (1.11–3.28); 0.019	G; (0.16 | 0.21); 1.42 (1.08–1.85); 0.011

rs6465657	7q21	*LMTK2 *	T; (0.18 | 0.14); 0.56 (0.36–0.87); 0.010	T; (0.18 | 0.23); 0.226	T; (0.18 | 0.17); 0.455

rs7008482	8q24	*NSMCE2 *	T; (0.44 | 0.31); **0.41 (0.26–0.62)**; 2.45 × 10^−5^	G; (0.42 | 0.42); 0.877	T; (0.51 | 0.60); **0.48 (0.34–0.68)**; 3.87 × 10^−5^

rs6983561	8q24	*PRNCR1 *	C; (0.16 | 0.18); 0.335	C; (0.06 | 0.10); 0.067	C; (0.11 | 0.15); 0.078

rs6983267	8q24	*POU5F1P1 *	T; (0.41 | 0.26); **0.35 (0.23–0.53)**; 4.48 × 10^−7^	T; (0.39 | 0.37); 0.774	T; (0.40 | 0.30); **0.53 (0.39–0.73)**; 8.05 × 10^−5^

rs4242382	8q24	*POU5F1P1 *	A; (0.24 | 0.19); 0.59 (0.39–0.89); 0.013	A; (0.20 | 0.19); 0.768	A; (0.22 | 0.19); 0.11

rs10993994	10q11	*MSMB *	T; (0.27 | 0.37); **1.56 (1.56–6.08)**; 1.40 × 10^−3^	T; (0.12 | 0.33); **3.49 (2.28–5.54)**; 1.56 × 10^−9^	T; (0.20 | 0.35); **2.09 (1.64–2.68)**; 3.03 × 10^−9^

rs4962416	10q26	*CTBP2 *	C; (0.49 | 0.46); 0.61 (0.36–0.97); 0.035	T; (0.43 | 0.47); 0.305	T; (0.48 | 0.46); 0.338

rs7931342	11q13	*MYEOV *	T; (0.44 | 0.38); 0.064	T; (0.48 | 0.48); 0.664	T; (0.46 | 0.41); 0.091

rs4430796	17q12	*HNF1B *	A; (0.44 | 0.39); 0.433	A; (0.48 | 0.44); 0.48 (0.26–0.84); 0.010	A; (0.46 | 0.45); 0.424

rs1859962	17q24	*CASC17 *	G; (0.28 | 0.28); 0.757	G; (0.29 | 0.36); 1.42 (1.01–2.01); 0.044	G; (0.28 | 0.30); 0.302

rs2735839	19q13	*KLK3 *	A; (0.31 | 0.36); 0.149	A; (0.36 | 0.34); 0.734	A; (0.33 | 0.35); 0.377

Only *p* < 1.47 × 10^−3^ (Bonferroni correction) indicated in bold text was considered statistically significant.

^a^OR (95% CI) only shown for SNPs where *p* < 0.05.

^b^Adjusted for age and ethnicity.

^‡^Genes indicated have been shown to be involved in prostate tumour onset or progression or to have altered expression in prostate tumours [34–41], except *NSMCE2*, shown to alter growth rates of breast cancer cells [42], *MYEOV*, shown to be overamplified in breast and oesophageal carcinomas [43], and *CASC17*, a long nonprotein coding RNA gene.
